# Risk of Cancer among Commercially Insured HIV-Infected Adults on Antiretroviral Therapy

**DOI:** 10.1155/2016/2138259

**Published:** 2016-11-02

**Authors:** Jeannette Y. Lee, Ishwori Dhakal, Corey Casper, Ariela Noy, Joel M. Palefsky, Missak Haigentz, Susan E. Krown, Richard F. Ambinder, Ronald T. Mitsuyasu

**Affiliations:** ^1^University of Arkansas for Medical Sciences, Little Rock, AR, USA; ^2^Duke University, Durham, NC, USA; ^3^University of Washington, Seattle, WA, USA; ^4^Memorial Sloan-Kettering Cancer Center, New York, NY, USA; ^5^University of California, San Francisco, San Francisco, CA, USA; ^6^Albert Einstein College of Medicine/Montefiore Medical Center, Bronx, NY, USA; ^7^AIDS Malignancy Consortium, New York, NY, USA; ^8^Johns Hopkins University, Baltimore, MD, USA; ^9^University of California, Los Angeles, Los Angeles, CA, USA

## Abstract

The objective of this study was to explore the cancer incidence rates among HIV-infected persons with commercial insurance who were on antiretroviral therapy and compare them with those rates in the general population. Paid health insurance claims for 63,221 individuals 18 years or older, with at least one claim with a diagnostic code for HIV and at least one filled prescription for an antiretroviral medication between January 1, 2006, and September 30, 2012, were obtained from the LifeLink® Health Plan Claims Database. The expected number of cancer cases in the general population for each gender-age group (<30, 30–39, 40–49, 50–59, and >60 years) was estimated using incidence rates from the Surveillance Epidemiology and End Results (SEER) program. Standardized incidence ratios (SIRs) were estimated using their 95% confidence intervals (CIs). Compared to the general population, incidence rates for HIV-infected adults were elevated (SIR, 95% CI) for Kaposi sarcoma (46.08; 38.74–48.94), non-Hodgkin lymphoma (4.22; 3.63–4.45), Hodgkin lymphoma (9.83; 7.45–10.84), and anal cancer (30.54; 25.62–32.46) and lower for colorectal cancer (0.69; 0.52–0.76), lung cancer (0.70; 0.54, 0.77), and prostate cancer (0.54; 0.45–0.58). Commercially insured, treated HIV-infected adults had elevated rates for infection-related cancers, but not for common non-AIDS defining cancers.

## 1. Introduction

With the widespread use of highly active antiretroviral therapy (HAART), the life expectancy of HIV-infected persons in the United States has increased markedly [1]. HIV-infected persons are at higher risk for cancer compared with the general population [2, 3]. Utilization of highly active antiretroviral therapy (HAART) has been associated with decreased incidence rates for AIDS-defining cancers (ADCs) among HIV-infected persons [4, 5], but its impact on non-AIDS-defining cancers (NADCs) has been mixed [6–8]. An elevated risk of anal cancer [9, 10] and Hodgkin lymphoma (HL) has been observed among HAART users [11, 12]. After adjusting for CD4+ cell count and HIV viral load, a relationship between duration of antiretroviral therapy and incidence of NADCs was not observed in a cohort study of HIV-infected adults [10].

In the general population, health insurance coverage has been associated with lower mortality rates [13] and higher utilization of cancer prevention measures such as screening procedures [14] and HPV vaccine uptake [15]. Among adults between 18 and 65 years of age in 2007, 67% had private insurance, mostly through their employers, 14% were covered by Medicaid, and 17% were uninsured [16]. Insurance coverage varied by educational level and geographic region [16]. Among adults, 28.0% of those who had not completed high school had private insurance compared with 53.9% with a high school degree or equivalent and 75.2% of those who pursued postsecondary education [16]. Private health coverage was reported by 60–62% of those in the West and South and 66–68% of those in the Midwest and East [16].

Reports vary on the health insurance coverage among HIV-infected persons. In the Medical Monitoring Project of HIV-infected persons who sought medical care in 2009, 81.1% had some insurance coverage and 30.6% were covered by private insurance over the preceding 12 months [17]. In a study of men who have sex with men (MSM) conducted in 21 metropolitan areas by the National HIV Behavioral Surveillance System, 75% of participants had current health insurance and 39% were solely covered by private insurance [18].

Among HIV-infected persons, factors associated with private insurance were white race, female gender, men who have sex with men, and having less than two CD4+ counts measures of ≤200 cells/mm^3^ in the preceding year [19]. Risk factors and receipt of health care are related to health insurance coverage among HIV-infected persons. Commercially insured patients initiated antiretroviral therapy at an earlier stage of their disease defined by CD4+ cell counts than those on Medicaid [20]. Inpatient hospitalizations occurred less frequently among HIV-infected patients with private insurance compared to those who were uninsured or who had other insurance [19]. HIV-infected MSM with private insurance were less likely to miss primary HIV care appointments than those without insurance [21].

While use of antiretroviral therapy and health care coverage are beneficial for the management of HIV, the implications are unclear with respect to development of cancer. The objective of this study was to explore the incidence of cancer among commercially insured HIV-infected individuals receiving antiretroviral therapy.

## 2. Materials and Methods

The source of the data for this study was the LifeLink® Health Plan Claims Database of paid insurance claims data from managed care plans and other sources in the United States. The full database includes information on over 73 million persons from over 80 commercial health care plans in the US. The database includes duration of participation on a health care plan from enrollment date or first claim date to termination date or last claim date, gender, age at enrollment, geographic region of residence, and insurance claims information. Geographic regions were defined based on the US Census Bureau Regions.

From this database, we obtained the paid health insurance claims for 65,341 individuals who had at least one claim with an ICD-9-CM diagnostic code for HIV (042, V08, or 079.53) and at least one filled prescription for an antiretroviral medication between January 1, 2006, and September 30, 2012. We excluded persons under 18 years of age, those for whom gender information was missing, and those for whom the initial claim date for HIV was after plan termination. Thus, 63,221 unique individuals were included in this analysis.

Cancer cases were identified based on claims in which at least one diagnostic code indicated cancer and the procedure performed was for cancer treatment with surgery, radiation therapy, or chemotherapy. Incident cases were defined as cancer cases diagnosed at least 60 days after the claim for antiretroviral treatment. If more than one cancer diagnosis was detected for an individual, this report includes the cancer diagnosis from the first claim date. Incidence rates are reported per 100,000 person-years.

Each individual's duration of risk from cancer was from the date of the initial antiretroviral claim until the termination of participation in the health plan. Duration of risk from cancer was further divided by attained age: <30, 30–39, 40–49, 50–59, or ≥60 years of age. For example, an individual who was 35 years of age at the initial HIV claim and terminated the health plan at 42 years of age would contribute 5 years to the cumulative duration of risk of those 30–39 years of age and 2 years to the cumulative duration of risk of those 40–49 years of age.

For AIDS-defining cancers (ADCs) and non-AIDS-defining cancers (NADCs) that occurred in at least 5% of the cancer cases, incidence rates per 100,000 person-years and their 95% Poisson confidence intervals were estimated. Poisson regression analyses were used to evaluate the association of age group and gender on incidence rates.

To determine whether cancer incidence rates among HIV-infected individuals differed from those for the general population, the expected number of cases for each gender-age group (<30, 30–39, 40–49, 50–59, and ≥60) were estimated using incidence rates from the Surveillance Epidemiology and End Results (SEER) program (http://seer.cancer.gov/). Standardized incidence ratios were estimated using their 95% confidence intervals. The Cochran-Armitage test was used to evaluate the trends for the proportion of cancers that were ADCs with age for each gender.

The study was reviewed by the Institutional Review Board (IRB) at the University of Arkansas for Medical Sciences. The IRB determined that this study was not human subjects research.

## 3. Results

This study included 50,960 men (80.6%) and 12,261 (19.4%) women. The mean (SD) ages at initial claim for antiretroviral therapy for men and women were 44.6 (9.6) years and 43.0 (9.8) years and their mean durations of participation in a health care plan following initial antiretroviral claim were 3.7 (3.2) years and 3.9 (3.5) years, respectively. The number of person-years at risk by gender and age group is summarized in Table 1.

**Figure 1:**
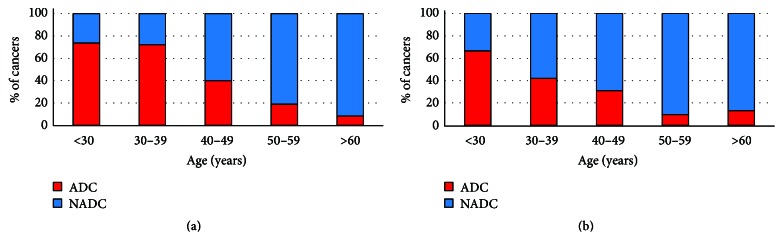
Distribution of AIDS-defining cancers (ADCS) and non-AIDS-defining cancers (NADCs) among men (a) and women (b).

A total of 1,130 persons had at least one treatment claim with a diagnosis of cancer: 978 men and 152 women. The most frequently reported cancers were non-Hodgkin lymphoma (NHL), Kaposi sarcoma (KS), anal cancer, and prostate cancer (Table 2). Overall, 28.9% of cancers were AIDS-defining. The proportion of cancers that were AIDS-defining decreased with age for both men (*P* < 0.001) and women (*P* < 0.001) (Figure 1). Among persons under 40 years of age, the majority of cancers were ADCs. After 60 years of age, over 90% of the cancers were NADCs.

The incidence of non-Hodgkin lymphoma did not vary by age or gender (Table 3). The incidence rates for Kaposi sarcoma decreased with age with lower rates for those 60 years and older compared to those 30–59 years of age (*P* = 0.038) which, in turn, were significantly lower than the incidence rates for persons under 30 years of age (*P* = 0.045). KS occurred more frequently among men (IR = 103.5) compared to women (IR = 12.9) (*P* < 0.001).

Anal cancer and Hodgkin lymphoma incidence rates varied by gender, but not by age (Table 4). Incidence rates for anal cancer were 93.6 and 45.0 for men and women, respectively (*P* = 0.011), and for Hodgkin lymphoma were 40.6 and 12.9, respectively (*P* = 0.025). HIV-infected persons 60 years or older had higher incidence rates than those 30–59 years of age for colorectal (*P* = 0.003), lung (*P* < 0.001), and prostate (*P* < 0.001) cancer.

As expected, incidence rates for KS and NHL were elevated compared to those in the general population and their SIRs declined with age. Incidence rates for anal cancer and Hodgkin lymphoma were elevated and varied by gender. SIRs for women and men were 15.66 (95% CI: 8.55 to 19.49) and 34.3 (95% CI: 28.46 to 36.56) for anal cancer, respectively, and 4.8 (95% CI: 1.28 to 7.56) and 10.7 (95% CI: 8.01 to 11.84) for Hodgkin lymphoma, respectively.

For colorectal, lung, and prostate cancer, incidence rates for HIV-infected persons were lower than those for the general population. For lung and colorectal cancer, the SIRs for persons 60 years and older were lower than those for individuals who were 30–59 years of age.

## 4. Discussion

From our examination of the incidence of cancer among privately insured HIV-infected adults in the US who were receiving antiretroviral therapy, we made several observations. We confirmed that infection-related cancers, including NHL, KS, and anal cancer, were the most commonly reported cancers in this population. Consistent with previous reports, we found that the incidence rates of noninfection related NADCs such as colorectal and prostate cancer were lower than those for the general population. Our finding that the incidence rate of lung cancer was lower than in the general population varies from previous reports. The proportion of cancer cases in this study that was ADCs was lower than previously reported [22–24]. Among HIV-infected cancer cases, about half were ADCs in an Italian study [22], one-third were ADCs in a single institution study in the US [23], and 60% were ADCs in an Australian observational database [24]. Our finding of the decline with age in the proportion of cancers that are ADCs is consistent with reports on the age-related decline in cancers attributable to infectious agents [25, 26].

Incidence rates for KS in this study were similar to those reported in other studies during the HAART era [4, 8]. Decreases in the incidence rate of KS have been observed with the increasing use of HAART [6, 27, 28] and increases in CD4+ cell counts [29, 30]. The increased risk of KS among men was related to the higher prevalence of human herpesvirus 8 infection, the etiologic agent of KS, among MSM compared to other risk groups [30, 31]. The declining incidence of KS associated with age observed in this study has been described previously [32].

The incidence of NHL observed in this study was similar to or lower than reported previously [4, 8]. Although a gender disparity in NHL incidence was not observed in this study, other reports showed that HIV-infected MSM have the highest risk of NHL, followed by other men and women [30]. The risk of NHL was inversely correlated with CD4+ cell count [29, 30]. Like KS, the incidence of NHL has decreased with time [6, 27, 28]. Although the incidence rates of both NHL and KS have decreased with time [27]. HIV-infected persons remain at elevated risk for both of these AIDS-defining cancers.

Our HL incidence was similar to previous reports [8, 33, 34]. The gender difference in HL incidence is similar to that reported in the general population [35]. There are mixed reports on the association between CD4+ cell counts and risk of HL among HIV-infected persons. The incidence of HL was positively correlated with CD4+ cell counts which suggests that improvements in CD4+ lymphocyte counts related to HAART use lead to an increasing risk of HL [33, 36]. Lower CD4+ lymphocyte counts in the 1-2 years preceding HL diagnosis were significantly associated with an elevated HL risk in the Swiss HIV Cohort Study [37]. It has been reported that HL incidence is highest in the 1–3 months following HAART initiation and decreases but remains elevated 4–6 months following HAART initiation [36]. Our finding that HL incidence among HIV-infected persons was elevated compared to the general population corroborates previous reports [2, 6, 33, 38].

Our anal cancer incidence rate for men was between the rates for MSM and other men reported in the North American (NA) ACCORD study [39] but lower than those from other reports [40–42]. Anal cancer incidence in women was similar to that in the NA-ACCORD study [39]. Anal cancer incidence rates among HIV-infected persons have increased with time [27, 28]. The SIR in this study was similar to that reported previously [2, 6, 8].

Human papillomavirus (HPV) infection is causally related to anal cancer. Among HIV-infected MSM, the prevalence of anal HPV is 77% and was lower among those on HAART [43]. Anal HPV prevalence in the general population was 12.9% [44]. Among MSM, detection of anal HPV was associated with receptive anal intercourse and number of male sexual partners in the preceding 6 months [45]. Among HIV-infected men, the prevalence of anal HPV-16, one of the high risk HPV types, decreases with educational level [46]. In a study of MSM, those with health insurance were less likely to engage in unprotected anal intercourse with male partners and had higher educational attainment than their uninsured counterparts [47]. Since our participant population was insured, the rate of anal cancer in men may reflect a lower prevalence of anal HPV due to less risky sexual behavior and higher educational attainment.

In our study, risks for cancers that are not infection-related were not elevated compared to the general population. The observed incidence of colorectal cancer does not differ significantly from previous reports [29, 41]. The SIR was consistent with that reported in a meta-analysis [2].

The lung cancer incidence rate among those 30–59 years old was lower compared to their older counterparts, consistent with the increasing risk of lung cancer with age in the general population [48]. Compared to the general population, the incidence rate in the younger age group did not differ significantly, but older HIV-infected persons had a lower lung cancer incidence rate.

Our findings vary from reports of an elevated risk of lung cancer associated with HIV [2, 5, 6, 8, 40, 41]. It has been reported that HIV patients are not at elevated risk after adjusting for smoking and other risk factors [29], but other reports show that HIV is an independent risk factor for lung cancer [5, 49]. HIV-infected persons are diagnosed with lung cancer at earlier ages and at lower cumulative smoking exposures than the general population [48, 50]. The decreased incidence of lung cancer in this study may reflect a lower prevalence of smoking in our population. In a study of MSM that measured health access using three indicators based on primary care clinic visits in the preceding 2 years, current health insurance, and perceived barriers to health care, smoking prevalence decreased with the increase in positive health access indices [51]. Among adults in the US, the proportion with private health insurance coverage increases with educational level [16] and smoking prevalence declines with educational level [52].

Reports on the role of immunosuppression in the incidence of lung cancer are mixed. A report from the French HIV Database Study reported an inverse relationship between lung cancer risk and CD4 counts [30] and an elevated risk of lung cancer was observed among HIV-infected individuals with CD4+ cells counts below 200 *μ*L in an integrated health care system in the US [29]. Other studies did not show a correlation between CD4 count and lung cancer risk [53, 54].

Our finding that prostate cancer incidence was reduced in HIV-infected men confirms previous reports [55, 56]. Although it was suggested that the decreased incidence rate was associated with a lowered use of the prostate-specific antigen (PSA) test to screen for prostate cancer [55], a comparison of HIV-infected and HIV-uninfected men in California found no difference with respect to PSA screening utilization [56].

As our case definition required an insurance claim for cancer treatment, cancer cases that were not treated were not included which may have underestimated the number of cancer cases. It has been widely reported that HIV-infected persons are less likely to receive treatment for cancer than their immunocompetent counterparts [57–59]. The disparity in receipt of treatment persists even among privately insured cancer patients [57]. Among lung cancer patients with private insurance, 23% of those infected with HIV and only 10% of those who were uninfected with HIV were untreated [57].

This study has a number of limitations. Since the database is one of administrative claims, the actual date of diagnosis was unknown. Our use of the 60-day window between initiation of antiretroviral use and cancer diagnosis may have led to an undercounting of cancer cases. Individual level information was not available for race/ethnicity, socioeconomic status, education level, level of immune suppression, or measures of sexual activity. The database did not allow evaluation of the relationship between HAART, markers of HIV disease, and cancer incidence.

This study in a population of commercially insured HIV-infected persons treated with antiretroviral therapy found elevated incidence rates for KS and NHL and infection-related NADCs, anal cancer, and Hodgkin lymphoma. Incidence rates for lung and colorectal cancer were not elevated in this HIV-infected population. Similar to other studies, prostate cancer incidence was lower among HIV-infected men compared to the general population. Although privately insured HIV-infected persons have greater access to health care services and earlier exposure to antiretroviral therapy (Schneider) and engage in fewer risky behaviors (McKirnan) than those with public insurance or who lack insurance, this did not appear to reduce the cancer incidence rates in this population.

## 5. Conclusions

Commercially insured HIV-infected adults treated with antiretroviral therapy were at greater risk of infection-related cancers compared to the general population. Their risk for common non-AIDS defining cancers of the lung, colon/rectum, and prostate was not elevated.

## Figures and Tables

**Table 1:** Person-years at risk by age group.

Age at risk (years)	Person-years at risk		
	Women Number of years (%)	Men Number of years (%)	Total Number of years (%)
<30	1752 (5.6)	5350 (4.1)	7102 (4.4)
30–39	7303 (23.5)	20775 (15.9)	28078 (17.4)
40–49	12140 (39.0)	55341 (42.4)	67481 (41.8)
50–59	7803 (25.1)	38303 (29.4)	46106 (28.5)
≥60	2111 (6.8)	10615 (8.1)	12726 (7.9)
Total	31109 (100.0)	130384 (100.0)	161493 (100.0)

**Table 2:** Cancer cases among HIV-infected persons.

Cancer site	Female *N* (%)	Male *N* (%)	Total *N* (%)
AIDS-defining cancer			
Kaposi's sarcoma	4 (2.6)	135 (13.8)	139 (12.3)
Non-Hodgkin's lymphoma	26 (17.1)	156 (16.0)	182 (16.1)
Cervix	5 (3.3)	—	5 (0.4)
Non-AIDS-defining cancer			
Oropharynx	1 (0.7)	21 (2.2)	22 (2.0)
Colon/rectum	6 (4.0)	56 (5.7)	62 (5.5)
Liver	3 (2.0)	13 (1.3)	16 (1.4)
Pancreas	2 (1.3)	13 (1.3)	15 (1.3)
Anus	14 (9.2)	122 (12.5)	136 (12.0)
Larynx	0	12 (1.2)	12 (1.1)
Lung, bronchus	12 (7.9)	51 (5.2)	63 (5.6)
Skin excluding melanoma, basal, and squamous cell	2 (1.3)	32 (3.3)	34 (3.0)
Breast	40 (26.3)	—	40 (3.5)
Bladder	4 (2.6)	24 (2.4)	28 (2.5)
Prostate	—	119 (12.2)	119 (10.5)
Hodgkin's lymphoma	4 (2.6)	53 (5.4)	57 (5.0)
Other	29 (19.1)	171 (17.5)	200 (17.7)
Total	152	978	1130

**Table 3:** Incidence rate for cancer in HIV-infected persons.

	<30 years	30–59 years	≥60 years	Total
AIDS-defining cancer				
Kaposi's sarcoma				
Male	168.2 (87.5, 323.3)	106.6 (89.3, 127.3)	37.7 (14.1, 100.4)	103.5 (67.5, 122.6)
Female	114.5 (28.6, 456.4)	7.3 (1.8, 29.4)	0 (0, 3.7)	12.9 (4.8, 34.3)
Both	154.9 (85.8, 279.7)	87.5 (73.4, 104.4)	31.4 (11.8, 83.7)	86.1 (72.9, 101.6)
Non-Hodgkin's lymphoma				
Male	94.5 (38.9, 224.5)	122.4 (103.7, 144.4)	103.6 (57.4, 187.1)	119.6 (102.3, 140.0)
Female	0 (0, 3.7)	84.4 (56.1, 127.0)	142.1 (45.8, 440.6)	83.6 (56.9, 122.8)
Both	70.4 (29.3, 169.1)	115.1 (98.7, 134.2)	110.0 (65.2, 185.8)	112.7 (97.5, 130.3)
Non-AIDS-defining cancer				
Colon/rectum				
Male	0 (0, 3.7)	39.3 (29.4, 52.7)	103.6 (57.4, 187.1)	43.0 (33.1, 55.8)
Female	0 (0, 3.7)	18.4 (7.6, 44.1)	47.4 (6.7, 336.3)	19.3 (8.7, 42.9)
Both	0 (0, 3.7)	35.3 (26.8, 45.6)	94.3 (53.6, 166.0)	38.4 (29.9, 49.2)
Anus				
Male	18.7 (2.6, 132.7)	94.4 (78.2, 114.0)	122.5 (71.1, 210.9)	93.6 (78.4, 111.7)
Female	0 (0, 3.7)	44.0 (25.0, 77.6)	94.7 (23.7, 378.8)	45.0 (26.7, 76.0)
Both	14.1 (2.0, 100.0)	84.7 (70.8, 101.3)	117.9 (71.1, 195.5)	84.2 (71.2, 99.6)
Lung, bronchus				
Male	0 (0, 3.7)	32.3 (23.4, 44.6)	131.9 (78.12, 222.7)	39.1 (29.7, 51.5)
Female	0 (0, 3.7)	36.7 (19.7, 68.2)	94.7 (23.7, 278.8)	38.6 (21.9, 67.9)
Both	0 (0, 3.7)	33.2 (24.9, 44.2)	125.7 (77.0, 205.2)	39.0 (30.5, 49.9)
Prostate	0 (0, 3.7)	54.2 (42.2, 69.5)	537.0 (414.2, 696.1)	91.3 (76.3, 109.2)
Hodgkin's lymphoma				
Male	74.8 (28.1, 199.2)	40.2 (30.1, 53.7)	28.3 (9.1, 87.6)	40.6 (31.1, 53.2)
Female	0 (0, 3.7)	14.7 (5.5, 39.1)	0 (0, 3.7)	12.9 (4.8, 34.3)
Both	56.3 (21.1, 150.1)	35.3 (26.8, 46.6)	33.6 (7.6, 73.1)	35.3 (27.2, 45.8)

**Table 4:** Standard incidence ratios (SIRs) for cancer in HIV-infected persons.

	<30 years	30–59 years	≥60 years	Total
AIDS-defining cancer				
Kaposi's sarcoma				
Male	280.37 (127.94, 372.43)	43.86 (36.42, 46.79)	23.55 (6.34, 37.39)	45.25 (37.94, 48.10)
Female	∞	82.37 (9.25, 165.59)	0	122.23 (32.89, 194.04)
Both	342.68 (170.83, 440.87)	44.20 (36.76, 47.12)	22.44 (6.04, 35.62)	46.09 (38.74, 48.94)
Non-Hodgkin's lymphoma				
Male	29.21 (9.41, 43.75)	5.14 (4.32, 5.45)	1.11 (0.55, 1.42)	4.17 (3.54, 4.42)
Female	0 (0, 0.65)	5.38 (3.41, 6.33)	2.11 (0.42, 3.66)	4.53 (2.96, 5.28)
Both	23.84 (7.68, 35.71)	5.17 (4.41, 5.46)	1.23 (0.67, 1.53)	4.22 (3.63, 4.45)
Non-AIDS-defining cancer				
Colon/rectum				
Male	0 (0, 0.42)	0.82 (0.60, 0.92)	0.50 (0.25, 0.65)	0.73 (0.55, 0.81)
Female	0 (0, 1.30)	0.52 (0.17, 0.78)	0.28 (0, 0.79)	0.45 (0.17, 0.65)
Both	0 (0, 0.32)	0.78 (0.58, 0.86)	0.47 (0.24, 0.60)	0.69 (0.53, 0.76)
Anus				
Male	∞	34.93 (28.65, 37.42)	27.83 (14.81, 34.97)	34.28 (28.46, 36.56)
Female	∞	15.64 (8.08, 19.87)	15.79 (1.77, 31.74)	15.66 (8.55, 19.49)
Both	∞	31.09 (25.78, 33.18)	25.26 (14.13, 31.16)	30.54 (25.62, 32.46)
Lung, bronchus				
Male	0 (0, 1.17)	0.85 (0.60, 0.96)	0.42 (0.23, 0.52)	0.66 (0.49, 0.74)
Female	0 (0, 4.72)	1.23 (0.59, 1.60)	0.41 (0.05, 0.83)	0.92 (0.48, 1.17)
Both	0 (0, 0.94)	0.91 (0.67, 1.01)	0.42 (0.24, 0.51)	0.70 (0.54, 0.77)
Prostate	∞	0.44 (0.33, 0.48)	0.74 (0.56, 0.82)	0.54 (0.45, 0.58)
Hodgkin's lymphoma				
Male	18.69 (5.03, 29.67)	10.81 (7.91, 12.07)	6.14 (1.23, 10.64)	10.69 (8.01, 11.84)
Female	0 (0, 0.34)	5.68 (1.53, 9.01)	0 (0, 0.39)	4.76 (1.28, 7.56)
Both	13.99 (3.76, 22.22)	10.08 (7.48, 11.20)	5.44 (1.09, 9.42)	9.83 (7.45, 10.84)
